# Pulmonary Involvement in Systemic Lupus Erythematosus: A Potentially Overlooked Condition

**DOI:** 10.3390/biomedicines13061485

**Published:** 2025-06-16

**Authors:** Ilaria Mormile, Gerardo Nazzaro, Marco Filippelli, Francesca Della Casa, Mauro Mormile, Amato de Paulis, Francesca Wanda Rossi

**Affiliations:** 1 Department of Translational Medical Sciences, University of Naples Federico II, 80131 Naples, Italy, ,; 2 Department of Clinical Medicine and Surgery, University of Naples Federico II, 80131 Naples, Italy; 3 Center for Basic and Clinical Immunology Research (CISI), WAO Center of Excellence, University of Naples Federico II, 80131 Naples, Italy

**Keywords:** autoimmunity, interstitial lung disease, systemic erythematous lupus, pleuritis, pulmonary involvement

## Abstract

Systemic lupus erythematosus (SLE) is a pleiotropic disease that can present in numerous forms, ranging from mild mucocutaneous symptoms to severe manifestations affecting multiple organs. SLE has the potential to impact any segment of the respiratory system, exhibiting a range of severity levels throughout the different stages of the disease. Pulmonary manifestations in SLE patients can be classified as primary (i.e., directly related to SLE and to immune-mediated damage), secondary to other SLE manifestations (e.g., nephrotic syndrome, renal failure, congestive heart failure), and comorbidities (e.g., infections, cancers, overlapping primary respiratory diseases). Understanding and correctly managing lung involvement in SLE is crucial because pulmonary complications are common and can significantly impact morbidity and mortality in affected patients. Early recognition and appropriate treatment can prevent irreversible lung damage, improve quality of life, and reduce the risk of life-threatening complications. Treatment algorithms are based on the suppression of inflammation, with or without the need for dedicated, supportive care. According to disease severity, available treatments include nonsteroidal anti-inflammatory drugs, corticosteroids, immunosuppressants, and biological agents. In this review, we aim to summarize the current knowledge on lung involvement in SLE and then focus on the management and treatment approaches available for the different forms.

## 1. Introduction

Systemic lupus erythematosus (SLE) is a complex condition influenced by multiple factors, including genetic predisposition, environmental influences, and dysregulation in both innate and adaptive immune responses [1]. SLE is a pleiotropic disease that can present in various forms, ranging from mild mucocutaneous symptoms to severe manifestations affecting multiple organs [2,3,4]. SLE can potentially impact any segment of the respiratory system through various pathogenic mechanisms, exhibiting a range of severity levels at different stages of the disease. Although most of the molecular mechanisms underlying lung involvement in SLE remain unknown, inflammation and immune response dysregulation are important drivers [5]. Indeed, potent inflammatory cytokines such as interleukin (IL)-6, IL-1, and IL-8; tumor necrosis factor (TNF)-α; and systemic type 1 interferon (IFN), which are overexpressed in SLE patients, together with circulating immune complexes and other unidentified mediators concur lung inflammation, tissue damage, vasculitis, and, ultimately, fibrosis [6,7,8,9,10]. Additionally, immune cells play a pivotal role in the pathogenesis of SLE, both in general and in its lung manifestations. Indeed, the lung inflammatory insult and the resulting cytokine milieu contribute to the recruitment of neutrophils, followed by monocytes, macrophages, and T and B lymphocytes, which participate in the pathogenesis in different ways [6,7,8]. Among the other actions, T cells activate B cells and contribute to lung tissue inflammation through the release of pro-inflammatory cytokines (e.g., IL-17, IFN-γ). In contrast, B cells contribute to the production of autoantibodies that form immune complexes, depositing in lung tissue and triggering inflammation and damage [11,12,13]. Neutrophils act through the NETosis process, in which they release DNA and histones, thereby revealing autoantigens and self-DNA, which further exacerbates inflammatory responses [14]. Macrophages engulf immune complexes and release inflammatory mediators (e.g., TNF-α, IL-6), promoting fibrosis and tissue injury [15,16]. These multifaceted immune responses lead to chronic inflammation, tissue remodeling, and vascular damage in the lungs, contributing to the diverse pulmonary manifestations of SLE.

Respiratory symptoms can manifest acutely and/or chronically, and often present as being nonspecific (e.g., cough, dyspnea, pleuritic chest pain) [6,17,18]. In addition, despite the presence of lung involvement, the patient can be asymptomatic [6,17,18]. Pulmonary manifestations in SLE patients can be classified as primary (i.e., directly related to SLE and to immune-mediated damage), secondary to other SLE manifestations (e.g., nephrotic syndrome, renal failure, congestive heart failure), and comorbidities (e.g., infections, cancers, overlapping primary respiratory diseases) [6,17,18]. Primary pulmonary manifestations in SLE can be further classified, according to the anatomical compartment involved (i.e., pleura, parenchyma, airways, pulmonary vasculature, and skeletal muscles), into pleuritis, interstitial pneumonitis, obliterative bronchiolitis, pulmonary arterial hypertension, and shrinking lung syndrome, respectively [6,17,18]. The prevalence of these manifestations may vary widely among different cohorts of patients due to different definitions and criteria used for establishing the diagnosis. However, pleural involvement is by far the most common manifestation (40–50%) [19,20]. Although less common than in other immune-mediated conditions [21,22,23,24], interstitial involvement is also possible, accounting for 2–4% of cases of acute interstitial pneumonitis and up to 4% of cases of chronic interstitial pneumonitis [17]. Pulmonary arterial hypertension has been reported in 2–8% of cases, while shrinking lung (1–2%) and obliterative bronchiolitis (<1%) are considered rare [25,26,27]. Diffuse alveolar hemorrhage (DAH) is considered rare. However, this condition is potentially life-threatening, being associated with a high mortality rate of up to 90% [18]. Also, acute interstitial pneumonitis may be fatal in up to 50% of cases [18]. In addition, although ILD is rare in SLE, it is a factor that affects the patient’s prognosis since it is a risk factor for reduced survival [28]. Understanding and correctly managing lung involvement in SLE is crucial because pulmonary complications are common and can significantly impact morbidity and mortality in affected patients. In this review, we aim to summarize the current knowledge on lung involvement in SLE and then focus on the management and treatment approaches available for the different forms.

## 2. Clinical Patterns of Pulmonary Involvement in Systemic Erythematous Lupus

### 2.1. Pleuritis

Pleural involvement can occur with or without fluid buildup and represents the most prevalent pulmonary manifestation associated with SLE. This manifestation typically occurs in patients with active SLE and may be unilateral or bilateral [20]. This inflammatory process is triggered by the infiltration of immune cells into the pleural space, resulting in the production of autoantibodies and inflammation [18,29,30].

Patients with SLE-related pleural involvement typically present with pleuritic chest pain, cough, fever, and dyspnea [18,31]. Additionally, some patients may develop a pleural effusion, which is often bilateral and exudative [18,29,32,33]. Interestingly, about 30–50% of SLE patients experience pleural effusion at some point during their disease course, although these effusions can be small and asymptomatic [18,32,34]. Medications such as hydralazine, procainamide, and anti-tumor necrosis factor-alpha agents can also induce pleuritis [18,35,36]. In such medication-induced cases, simply stopping the culprit drug often leads to remission [18,35,36].

**Figure 1:**
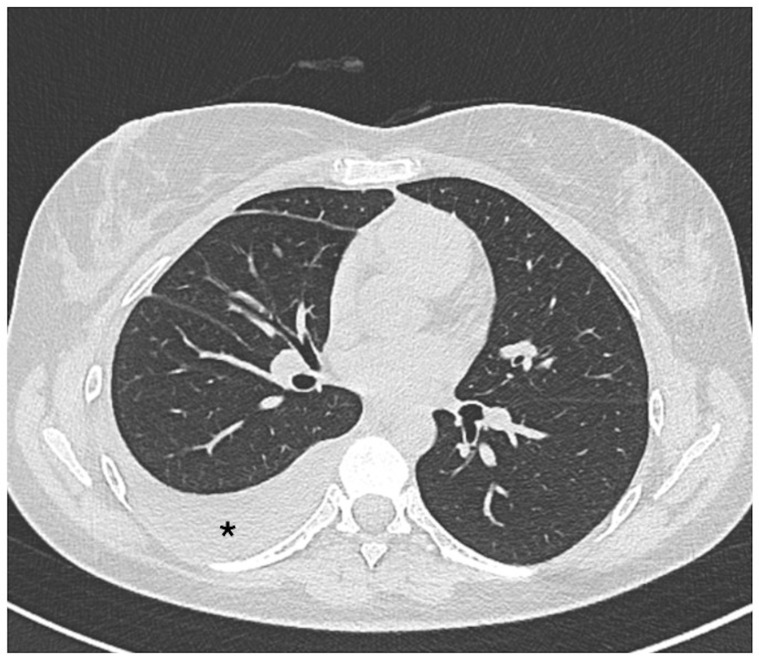
Axial non-contrast thorax computed tomography scan in a patient with systemic lupus erythematosus. The exam revealed abnormal accumulations of fluid within the pleural space (asterisk).

A chest X-ray showing effusion and/or thickening is usually sufficient to establish a diagnosis. In addition, it is crucial to rule out other potential causes of pleural inflammation that can occur alongside SLE, including infections, pulmonary embolism, cancers, heart failure, and pericarditis [29]. The pleural effusion can also be visualized on a chest CT scan (Figure 1).

**Figure 2:**
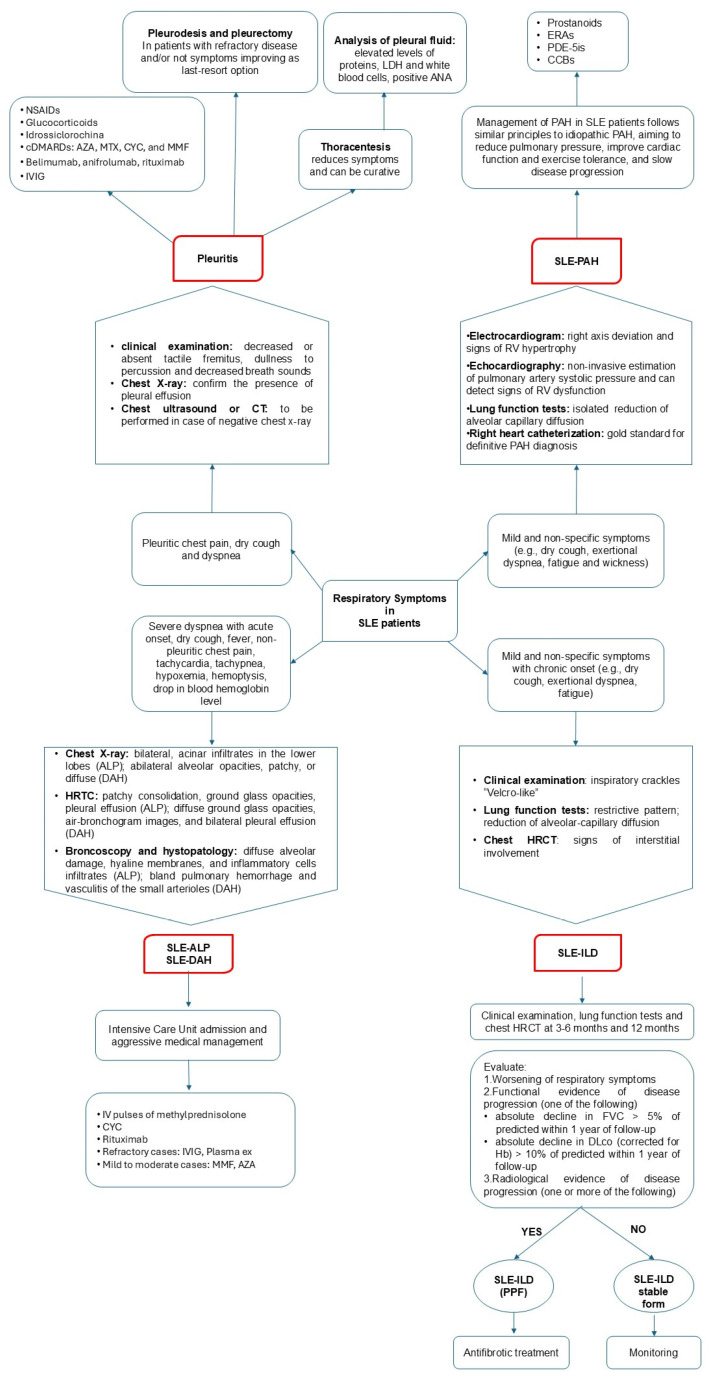
Diagnostic flowchart and suggested management for patients with systemic lupus erythematosus (SLE) presenting with respiratory symptoms. ALP, acute lupus pneumonitis; AZA, azathioprine; CYC, ciclophosphamide; CCBs, calcium channel blockers; CT, computed tomography; DAH, diffuse alveolar hemorrhage; ERAs, endothelin receptor antagonists; Hb, hemoglobin; HRTC, high-resolution computed tomography; ILD, interstitial lung disease; IVIG, intravenous immunoglobulin; MMF, mycophenolate mofetil; NSAIDs, nonsteroidal anti-inflammatory drugs; PAH, pulmonary arterial hypertension; PPF, progressive pulmonary fibrosis; PDE-5is: phosphodiesterase 5 inhibitors.

Pleural fluid analysis can be performed, even though it is often considered unnecessary unless the case is uncertain and infection is suspected [20]. Classically, pleural fluid in SLE patients demonstrates elevated protein levels, lactate dehydrogenase (LDH), and white blood cells. Additionally, some patients may exhibit a positive antinuclear antibody (ANA) test in the pleural fluid [29,30,32,37]. Figure 2 summarizes the proposed management for patients with SLE and respiratory symptoms.

**Figure 3:**
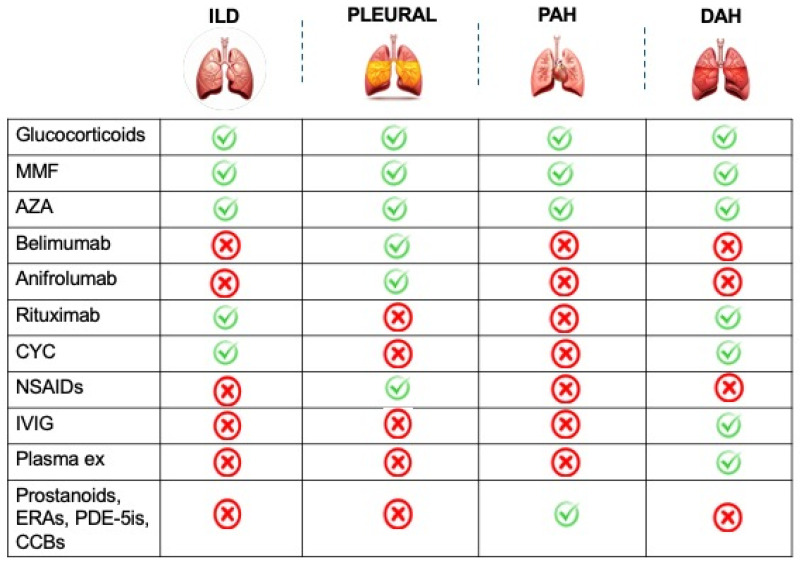
Treatment strategies available for pleuropulmonary involvement in systemic lupus erythematosus patients. AZA, azathioprine; CYC, cyclophosphamide; CCBs, calcium channel blockers; DAH, diffuse alveolar hemorrhage; ERAs, endothelin receptor antagonists; ILD, interstitial lung disease; IVIG, intravenous immunoglobulin; MMF, mycophenolate mofetil; NSAIDs, nonsteroidal anti-inflammatory drugs, PAH, pulmonary arterial hypertension; PDE-5is, phosphodiesterase 5 inhibitors.

The treatment strategies largely depend on the severity of symptoms (Figure 3).

Mild forms are managed with nonsteroidal anti-inflammatory drugs (NSAIDs), acting on inflammation and providing pain relief [31]. Long-term use of these drugs is problematic due to their gastrointestinal side effects and possible impact on kidney function [38]. Hydroxychloroquine can be added in these cases [39]. Corticosteroids are a cornerstone of therapy in patients with severe pleuritis and/or pleural effusion [40,41]. However, for patients with chronic serositis, due to the occurrence of side effects, steroid-sparing agents are often required. Several conventional Disease-Modifying Antirheumatic Drugs (cDMARDs) have shown effectiveness in reducing serosal involvement in SLE patients, including azathioprine (AZA), methotrexate (MTX), cyclophosphamide (CYC), and mycophenolate mofetil (MMF) [18,30,31,42]. Belimumab, a monoclonal antibody that inhibits the production of BAFF (B-cell-activating factor), was approved by the US Food and Drug Administration (FDA) in 2011 for SLE and more recently for lupus nephritis [43]. This drug has also been demonstrated to be effective in SLE-related pleuropericarditis [44]. Despite its efficacy, rituximab is currently an off-label therapy in patients with SLE. In a study by Ng et al. [45], two out of seven patients with lupus pleuritis experienced improvement after rituximab.

Occasionally, intravenous immunoglobulin (IVIg) has been used in cases of severe pleuritis [46] and massive pleural effusion [47] with promising outcomes, although its efficacy requires further validation.

Pleurodesis or pleurectomy represents a last-resort option for patients with refractory disease [48,49].

### 2.2. Lung Parenchymal Involvement

Lung parenchymal involvement is generally considered rare in SLE and can manifest in acute or chronic forms, each with distinct clinical presentations.

#### 2.2.1. Acute Clinical Presentation

SLE can rarely present with sudden lung complications like acute lupus pneumonitis (ALP) and diffuse alveolar hemorrhage (DAH) [29]. These forms are characterized by a sudden and often dramatic onset of symptoms, and, in severe cases, they can rapidly evolve into acute interstitial pneumonia (AIP) or acute respiratory distress syndrome (ARDS) [29,50]. These complications require immediate admission to the intensive care unit and aggressive medical management [51,52].

ALP is considered a rare complication in SLE patients, occurring in 1–3% of cases [17]. Although it is not common, this complication should not be overlooked due to a relatively high mortality associated with this event (up to 50%) and the possible development of chronic ILD in survivors [53]. It can occur as the presenting symptom of SLE [53], but it is more common during flares and in concomitance with multisystemic involvement [17]. In particular, an association between ALP and lupus nephritis has been described [54]. Another factor associated with the development of this severe complication is the presence of anti-Ro/SSA antibodies [54]. ALP symptoms and signs include acute dyspnea, fever, cough, tachycardia, tachypnea, hypoxemia, and basilar crackles [17]. Imaging tests are crucial for diagnostic assessment (Figure 2), even though chest X-ray findings are non-specific, while CT imaging (HRTC) is considered more sensitive. The first usually presents with bilateral patchy alveolar infiltrates, which may resemble infectious pneumonia, ARDS, or pulmonary edema, and pleural effusions in the absence of signs of volume overload, helping to differentiate ALP from cardiogenic pulmonary edema [55]. HRTC can reveal ground-glass opacities, consolidations, or interstitial infiltrates [55]. Diagnosis is usually established based on a combination of clinical and radiologic parameters, supported by bronchoalveolar lavage (BAL) and the exclusion of infectious pneumonia. Lung biopsy is recommended to confirm the diagnosis, showing alveolar wall damage, necrosis, edema, and hyaline membrane [17].

Treatment usually involves high-dose corticosteroids (1 mg/kg/day). For severe cases, additional immunosuppressive medications such as AZA, MMF, or CYC are usually needed (Figure 3) [54]. In refractory cases, intravenous immunoglobulins (IVIGs) and plasmapheresis may be considered [54].

With an estimated prevalence of 2%, DAH is a rare but potentially life-threatening manifestation of SLE, showing a mortality which can reach 90% in some series [56]. Indeed, DAH can lead to respiratory failure if not recognized and treated promptly. DAH is relatively uncommon as a presenting symptom of SLE, as it typically arises during the disease course in patients with clinical and serological markers of active disease [57,58]. For example, DAH is more common in patients with lupus nephritis, thrombocytopenia, elevation of CRP, and positivity of anti-Ro/SSA and antiphospholipid antibodies [59,60,61,62]. In particular, in SLE patients, DAH and thrombocytopenia can be closely associated. Indeed, these two manifestations are not only the clinical expression of the same underlying disease, but thrombocytopenia can represent a significant contributing factor to DAH development [63,64]. When alveolar capillaries are inflamed or damaged, as in some SLE patients, the increased bleeding risk linked to thrombocytopenia can tip the balance toward hemorrhage [63,64]. This clinical scenario may occur in SLE patients with or without the presence of antiphospholipid antibodies. However, a study by Figueroa-Parra et al. [64], which combined a multicenter cohort with a systematic literature review, found that antiphospholipid-syndrome-associated DAH is associated with high morbidity and mortality, particularly when presenting with triple positivity, thrombocytopenia, valvular involvement, and catastrophic antiphospholipid syndrome. DAH pathogenesis involves the formation of immune complexes (antibody–antigen complexes) that can deposit in various tissues, including the lung capillaries [51]. This deposition can lead to inflammation and damage to the blood vessels of the lungs, causing bleeding [51]. The resulting vasculitic process can compromise the integrity of the blood vessels, leading to the leakage of blood into the alveolar spaces. In addition, the cytokine storm observed in patients with active SLE can also contribute to capillary damage and hemorrhage in the lungs [51]. DAH signs and symptoms include dyspnea, dry or productive cough, hemoptysis, hypoxemia, fever, fatigue, general signs of systemic inflammation, and chest pain (mainly due to pleuritic involvement accompanying the bleeding and inflammation in the lungs) [17]. The diagnosis is suggested by the combination of clinical presentation in the context of a drop in blood hemoglobin levels with radiological findings (Figure 2) [17]. A chest X-ray may show bilateral infiltrates or consolidations, but these findings are nonspecific and can mimic other conditions, such as infectious pneumonia or pulmonary edema [51]. A CT scan is more sensitive than X-rays. Common findings include ground-glass opacities, consolidation, or a crazy-paving pattern (interlobular septal thickening with ground-glass opacities) [17,51]. Due to the presence of extravascular hemoglobin within the alveoli, the diffusion capacity of the lungs for carbon monoxide (DLCO) is typically increased [17]. BAL is crucial to the differential diagnosis when DAH could be associated with infection (viral, fungal, and others) because the clinical criteria are adequate to support the DAH diagnosis (in particular, >2 g hemoglobin decrease, hypoxia, dyspnea, in addition to imaging studies characteristically showing alveolar filling in HRCT), in spite of hemoptysis absence. In addition, elevated red blood cells in the lavage fluid are indicative of DAH [17,51]. Finally, in cases where the diagnosis is unclear, a lung biopsy can help confirm the presence of alveolar hemorrhage and show other features, such as immune complex deposition, necrotizing vasculitis, and hemosiderin-laden macrophages [65]. Treatment of DAH in lupus is centered on supportive care (e.g., fluid management, blood transfusion), oxygen therapy, and mechanical ventilation, as well as addressing the underlying lupus activity (Figure 3). High-dose corticosteroids and intravenous methylprednisolone pulse therapy are often the first line of treatment. CYC and rituximab may be used in severe cases, while MMF and AZA may be used in mild to moderate cases. Plasmapheresis may be considered in certain cases, especially if there is evidence of significant immune complex involvement or Goodpasture syndrome [17,51].

#### 2.2.2. Chronic Clinical Presentation

It appears that interstitial lung disease (ILD) affects a relatively small percentage of SLE patients, estimated to be between 2% and 4% [66,67,68,69]. Nevertheless, it is worth noting that a higher prevalence has been observed in certain cohorts. For instance, a study from Japan reported a prevalence as high as 29% in a cohort of hospitalized patients suffering from SLE [70]. Interestingly, ILD manifestations were present in many cases at the time of SLE diagnosis [66,67,70]. However, other reports suggest that lung involvement manifests later in the disease course and reveal that late-onset SLE patients are more prone to pulmonary manifestations than younger patients, possibly due to age-related immune senescence of the lungs [7,67,71]. Indeed, older age has also been linked to a higher likelihood of developing ILD, with a prevalence of up to 30% found in SLE patients over 50 years old [66,67,70,72]. Regarding the risk factors associated with ILD development, both age and duration of autoimmune disease have been implicated. One study found an average of 7.7 years between the onset of SLE and the development of ILD [66,67,69]. In addition, in SLE patients, ILD is often associated with another systemic autoimmune disorder such as mixed connective tissue disease, Sjögren’s syndrome, and inflammatory myopathies [28]. Interestingly, the severity of ILD does not necessarily correlate with the presence of SLE serologic markers [54,73].

**Figure 4:**
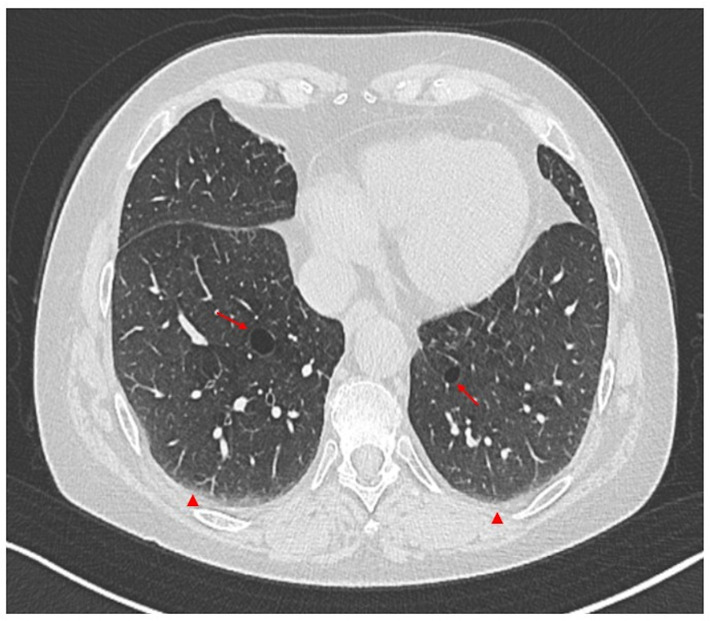
Axial non-contrast thorax computed tomography scan in a patient with systemic lupus erythematosus (SLE). The exam revealed abnormal permanent enlargement of the airspaces distal to the terminal bronchioles (arrows), accompanied by the destruction of the alveolar wall and subpleural interstitial involvement (arrowheads).

ILD in SLE usually runs asymptomatically, and this finding is incidental [29,50,54,73]. However, in other cases, symptoms may be initially mild and nonspecific, including dry cough, exertional dyspnea, and fatigue [18,38]. A physical exam may reveal inspiratory crackles during breathing, although fingernail deformities (clubbing) are uncommon. Lung function tests often reveal a restrictive pattern, characterized by a decrease in the DLCO (Figure 2) [29,73]. Le Tallec et al. [74] demonstrated a significantly higher prevalence of impaired DLCO among SLE patients, corroborating previous findings. They observed a strong association between reduced DLCO and severe clinical manifestations, including pleuritis, lymphadenopathy, and renal involvement. These results suggest that compromised DLCO may reflect widespread lung damage and be an early indicator of interstitial lung disease [74]. In diagnosing lung disease, HRCT plays a crucial role in identifying the lung involvement and characterizing the specific disease pattern (Figure 4).

Studies reveal non-specific interstitial pneumonia (NSIP) to be the most common pattern observed on HRCT. However, usual interstitial pneumonia (UIP) is also observed in a significant number of cases [67,70,73]. The most frequent abnormal findings include ground-glass opacities, consolidation, honeycombing, and traction bronchiectasis [67,68,73].

Early and accurate diagnosis of ILD in SLE allows for appropriate treatment, the careful monitoring of disease progression, improved patient prognosis, and enhanced quality of life [67,73]. While current treatment modalities for ILD-SLE offer some relief, they primarily focus on slowing disease progression rather than achieving complete remission [36]. This highlights the urgent need for a more precise medicine approach that tailors therapy to individual patient needs and disease severity. Immunosuppressive agents, such as CYC or MMF, can be employed, drawing insights from their success in treating scleroderma-associated ILD (Figure 3) [2,66,67,75]. Rituximab, another potential weapon in the therapeutic arsenal, may prove valuable as second-line therapy, particularly in severe and progressive cases [67,76,77,78]. Studies suggest it may even prevent the need for lung transplantation [79]. Emerging biological therapies, such as belimumab and anifrolumab, offer a promising avenue for future treatment; however, additional research is required to explore their efficacy and safety profiles in ILD-SLE [67,76]. In severe cases that do not respond to other treatments, IVIG and plasmapheresis can be used [67,76]. For patients who have progressive ILD secondary to SLE, anti-fibrotic medications (e.g., nintedanib) may be considered [66,67].

Focusing on precision medicine, addressing knowledge gaps, and exploring novel therapeutic options can create a brighter future for ILD-SLE patients, offering them the prospect of improved disease control and a better quality of life.

### 2.3. Bronchiolitis Obliterans Organizing Pneumonia

Bronchiolitis obliterans organizing pneumonia (BOOP) is a rare pulmonary disorder characterized by the formation of fibrous tissue plugs in the small airways and alveoli [80,81]. This condition has been linked to several factors, such as infections, inhaled toxins, drugs, and connective tissue diseases [82]. BOOP is considered an inflammatory rather than a fibrotic process and has been linked to connective tissue disorders, including rheumatoid arthritis and SLE [80,81,83]. Although rare in SLE, BOOP should be considered in SLE patients with respiratory symptoms such as dyspnea on exertion and dry cough [81,84,85]. Lung biopsy remains the gold standard for diagnosing BOOP; however, conventional radiography and CT scans can also reveal bilateral and diffuse alveolar infiltrates, particularly in the lower lobes [86]. BOOP patients typically respond well to systemic steroid treatment, resulting in prompt improvement and recovery.

### 2.4. Pulmonary Hypertension

Pulmonary arterial hypertension (PAH) is a severe and progressive pulmonary vascular disorder and represents a significant complication of SLE. The prevalence of this condition in SLE patients has been explored by several research groups and summarized in a meta-analysis by Lv et al. [26]. The authors found that the pooled prevalence of PAH in SLE patients was 8%, but also stressed that the prevalence of PAH differed significantly in the different cohorts based on different genders, ages, regions, years of publication, and diagnostic methods [26].

The underlying pathophysiological mechanisms contributing to PAH in SLE are multifaceted and involve a complex interplay of factors. The increased pulmonary pressure could be a delayed consequence of the cardiopulmonary involvement of SLE (such as left ventricular dysfunction and congestive heart failure), parenchymal lung diseases, and chronic hypoxemia due to SLE-related lung involvement (e.g., ILD) [18,54,87]. However, it could also involve immune-mediated mechanisms, including pulmonary vascular endothelium injury mediated by autoantibodies (e.g., anti-endothelial antibodies), immunocomplexes, complement activation, inflammatory cytokines (e.g., IL-6 and TNF-α), vascular remodeling (i.e., smooth muscle proliferation, fibrosis, and plexiform lesions), and thrombosis in situ (especially in patients with antiphospholipid antibodies) [88,89,90,91,92]. Indeed, in SLE patients, PAH can be classified as precapillary pulmonary hypertension resulting from pulmonary vasculitis, ILD, or chronic thromboembolism (especially with antiphospholipid syndrome), and postcapillary pulmonary hypertension resulting from cardiac involvement such as myocarditis or pericardial disease [93,94].

PAH’s clinical presentation in SLE patients can be subtle and nonspecific, often mimicking other comorbidities associated with the disease. For example, frequent symptoms include generalized fatigue and weakness, which are, in general, common features of SLE [18,54]. Other symptoms include chest pain and dyspnea, particularly worsening with exertion, which is a cardinal feature of PAH and may reflect impaired respiratory function [18,54].

Diagnosis of PAH in SLE patients requires a meticulous approach that integrates clinical evaluation, instrumental assessments, and more invasive investigations (Figure 2). Electrocardiogram findings may suggest right ventricular hypertrophy and right axis deviation, indicating increased pulmonary pressure [89,95]. Chest CT provides detailed images of the lungs and pulmonary vasculature, allowing for a more accurate assessment of PAH etiopathology [89,95]. Echocardiography provides a non-invasive estimation of pulmonary artery systolic pressure and can detect signs of right ventricular dysfunction [89,95]. Pulmonary function tests may show an isolated reduction in DLCO. Finally, right heart catheterization is considered the gold standard for the definitive diagnosis of PAH [54,96,97]. This invasive procedure directly measures pressure within the pulmonary arteries and assesses blood flow and pulmonary vascular resistance [54,96,97].

The management of PAH in SLE patients follows similar principles to those in idiopathic PAH, aiming to reduce pulmonary pressure, improve cardiac function and exercise tolerance, and slow disease progression [18]. There are several therapeutic options (Figure 3). Prostacyclin analogs mimic the effects of prostacyclin. Examples include epoprostenol, iloprost, treprostinil, and selexipag [73,98]. Endothelin receptor antagonists (ERAs) block the action of endothelin-1. Examples include bosentan, macitentan, and ambrisentan [73,98,99]. Phosphodiesterase 5 inhibitors (PDE-5Is) increase intracellular levels of cyclic adenosine monophosphate (cAMP). Examples include sildenafil and tadalafil [73,98]. Guanylate cyclase stimulants enhance the production of cyclic guanosine monophosphate (cGMP). An example is riociguat [4,36]. Calcium channel blockers (CCBs) can be beneficial in selected patients who demonstrate a positive response to acute vasodilator testing [73,98]. For patients with severe or refractory PAH, a combination therapy approach involving two or more different drug classes may be considered. This strategy aims to achieve a more comprehensive therapeutic effect and potentially improve outcomes [54,73,98,100,101,102]. Some studies suggest a potential role for immunosuppressive medications, particularly CYC with or without glucocorticoids, in treating SLE-associated PAH. Other immunosuppressants such as rituximab, MMF, and cyclosporine have also been explored in small studies [54,73,101,103]. Diuretics, anticoagulants, and oxygen therapy may be implemented to manage specific symptoms and improve overall patient well-being [54,73,102,103]. Despite advances in treatment, the prognosis for PAH in SLE patients remains challenging, with mortality rates still significantly higher than in the general population [88].

### 2.5. Shrinking Lung Syndrome

Shrinking lung syndrome (SLS) is a rare complication of SLE affecting approximately 1% of patients [5,30,104]. SLS is characterized by a reduction in lung volume, evidenced by an elevated hemidiaphragm on chest radiography and a restrictive pattern on pulmonary function tests without evidence of interstitial lung disease or significant structural lung abnormalities on imaging [5,30,104]. SLS clinical presentation is nonspecific, including respiratory symptoms such as dyspnea (mainly exertional), chest pain, and dry cough. Therefore, differentiating SLS from other conditions that cause reduced lung volumes, like pulmonary fibrosis, obesity, diaphragmatic paralysis, and central nervous system disorders, is crucial [104].

The pathophysiology of SLS remains incompletely understood. Initially, it was proposed that microatelectasis due to surfactant deficiency could be involved. Further research has explored various other potential mechanisms. Respiratory muscle weakness has been investigated but not confirmed by phrenic nerve stimulation and creatine kinase (CK) level assessments [104,105,106,107]. Steroid-induced myopathy is unlikely, as SLS can occur without prior steroid use. Diaphragmatic fibrosis and phrenic nerve palsy have been considered, although normal phrenic nerve conduction is demonstrated in most cases [104,108,109]. Since pleural adhesions have been observed in SLS, it has been suggested that pleural effusion or inflammation might contribute to limited diaphragmatic movement, given the prominence of pleuritic chest pain [104,105,110,111]. In addition, pleural inflammation could inhibit deep inspiration via neural reflexes, leading to chronic lung hypo-inflation, parenchymal remodeling, and decreased lung compliance, potentially creating a feedback loop [104,112].

While a definitive treatment for SLS remains elusive, corticosteroids are the most frequently employed therapeutic approach. Beyond corticosteroids, certain immunosuppressants, such as AZA, MTX, hydroxychloroquine, and cCYC, have been utilized in some SLS cases [113,114]. Some case reports have described the successful use of rituximab in SLE-related SLS in both adult [115,116] and pediatric cases [117,118]. Although the complete normalization of lung function is less common (<20% of patients), the long-term prognosis for SLS is generally favorable, with most patients experiencing clinical and functional improvement [119].

### 2.6. Infections

Patients with SLE have a significantly increased risk of infections compared to the general population due to immune system dysfunction, organ damage, and the use of immunosuppressive medications [29,120,121,122,123,124,125,126].

Immune system dysfunction is characterized by defects in both innate and adaptive immunity, as well as the presence of autoantibodies. In addition, the co-occurrence of SLE and primary immunodeficiencies has been described [122,127,128]. For example, genetic variations in mannose-binding lectin, a protein that helps the immune system recognize and eliminate threats, can lead to a more severe form of autoimmune disease like SLE and an increased risk of infections, especially those affecting the respiratory system [122,129].

Bacterial infections are the most common type of infection in SLE patients, frequently affecting the respiratory tract, where Streptococcus pneumoniae is the most frequently involved pathogen [122,130,131]. Viral infections are also common in SLE patients, including herpes zoster and cytomegalovirus infections [122].

Although opportunistic infections are generally less common than typical bacterial and viral infections, when they occur, they pose a significant threat due to their high morbidity and mortality rates. In particular, SLE patients have a 24-fold higher risk of developing an opportunistic infection than the general population [122,132]. Invasive fungal infections, which are among the most serious opportunistic infections, occur more frequently within the first year of SLE diagnosis. Major risk factors include high-dose glucocorticoid use (>60 mg/day) and high disease activity [122,133]. Pneumocystis jiroveci pneumonia (PJP), while rare, can be fatal in SLE patients, especially those with significant immunosuppression. Despite its severity, there are no clear guidelines on PJP prophylaxis, and clinical practice varies widely [122,134]. Recent studies suggest that universal prophylaxis is not recommended due to the low incidence of PJP and potential side effects in SLE patients [122].

Tuberculosis is another concern for SLE patients, since they present a higher risk of contracting the infection than the general population, and the disease tends to have a more severe course, with higher relapse rates and extrapulmonary involvement [122]. Additionally, glucocorticoid use, common in SLE treatment, is associated with increased mortality during tuberculosis treatment [122,135].

SLE patients have a slightly higher risk of contracting COVID-19 infection than the general population [136,137]. Furthermore, SLE patients are more likely to experience severe COVID-19 outcomes, including hospitalization, intensive care unit admission, and death, compared to the general population [132,136,138,139]. Several factors increase the risk of severe COVID-19 in SLE patients, including older age, male sex, underlying comorbidities, and the use of certain medications for SLE treatment, such as high-dose glucocorticoids and recent CYC therapy [136,140,141].

Managing infections in SLE patients is a complex challenge requiring an individualized approach. Understanding the different types of infections, associated risk factors, and potential complications is crucial. Better tools are needed to stratify individual risk and optimize preventive strategies to improve these patients’ outcomes and quality of life. Hydroxychloroquine, an antimalarial medication, has been demonstrated to decrease the risk of infections in patients with SLE. The exact mechanism by which hydroxychloroquine achieves this effect is not fully understood; however, it is thought to be due to its ability to modulate the immune response and reduce inflammation [122,142,143]. In addition to hydroxychloroquine, several other strategies can be used to prevent and treat infections in SLE patients. These include prophylactic antibiotics, vaccinations, and intravenous immunoglobulins [122,144]. The choice of preventive strategy or treatment should be individualized for each patient, based on their risk of infection and other factors.

### 2.7. Chronic Obstructive Pulmonary Disease

Increasing evidence suggests an association between autoimmune disease and the development of chronic obstructive pulmonary disease (COPD), since the role of autoimmunity in the development of the latter is becoming increasingly appreciated [145,146]. Using data from the Taiwan National Health Insurance Research Database (NHIRD), Shen TC et al. [146] found that the overall incidence rate of COPD was 1.73-fold higher in the SLE cohort than in the control cohort. More recently, Katz et al. [147] evaluated the prevalence of asthma and COPD in SLE cohorts, analyzing data from two large longitudinal cohorts of SLE patients, the Forward Lupus Cohort (FORWARD) and the Lupus Observation Study (LOS). The prevalence of asthma and COPD was assessed at baseline, showing that nearly 20% of FORWARD cohort participants reported asthma, while approximately 8% reported COPD [147]. Additionally, LOS cohort data revealed that 36% of participants had at least one of the two conditions. These prevalences are significantly higher than those in the general population, suggesting an association between SLE and an increased risk of asthma and COPD [147]. As a comorbidity, COPD is also notable since it represents a significant cause of increased hospitalization in SLE patients, as described by Han GM et al. [148]. Indeed, the authors reported that COPD accounted for 6.9% and 3.7% of hospitalizations in males and females aged 60 years and over, respectively, and was also a leading cause of emergency room visits (6.3% in men over 60 years and 2.4% in women in the age group of 40–59 with SLE). The mechanisms underlying the increased prevalence of asthma and COPD in SLE are not fully understood, and further research is needed to investigate potential shared risk factors and pathogenic pathways.

## 3. Predictive Factor of Lung Involvement in Systemic Lupus Erythematosus

The literature about the predictive factors of lung involvement in SLE is currently limited. However, Alamoudi et al. reported the correlation between abnormalities in the lungs detected by high-resolution computed tomography (HRCT) scans and hypocomplementemia, high levels of anti-dsDNA antibodies, and active disease, suggesting a potential correlation between these findings and lung involvement in SLE patients from Western Saudi Arabia [149]. In addition, several studies hypothesized that the serum biomarker KL-6 may predict the development of ILD even before it is visible on chest HRCT [150,151,152], even in juvenile SLE [153]. A Polish study investigated the complex relationship between iron metabolism and cytokine profiles in SLE patients [154]. They discovered a compelling association between elevated levels of soluble transferrin receptor (sTfR), a marker of iron deficiency or increased erythropoiesis, and the development of a range of organ manifestations, including pulmonary complications [154]. These findings underscore the potential significance of iron dysregulation in the pathogenesis of SLE [154].

In addition, other systemic manifestations of SLE can influence the development of lung involvement. Cytopenias are a frequent manifestation of SLE and can affect all blood cells, although the most common forms are lymphopenia, anemia, and thrombocytopenia [155]. There are different etiologies for these manifestations, ranging from the iatrogenic form induced by immunosuppressive drugs such as MTX or MMF to immune-mediated (e.g., autoimmune hemolytic anemia, immune thrombocytopenia, autoimmune neutropenia) and disease-related (non-immune) mechanisms such as the bone marrow function impairment cause by chronic inflammation or lupus itself [155,156,157]. Although they can also occur independently, cytopenias and lung involvement are interconnected in several clinical and pathophysiologic ways in SLE. Indeed, both cytopenias and lung involvement are markers of active or severe SLE [158]. Moreover, thrombocytopenia can worsen bleeding risk in DAH, which can in turn cause anemia [63,64]. Notably, cytopenias (especially leukopenia/neutropenia) are a significant risk factor for pulmonary infections, including opportunistic infections (e.g., Pneumocystis jirovecii, Mycobacterium tuberculosis, fungal) [159]. Finally, some immunosuppressants used for SLE, such as CYC and MTX, have been associated with both lung and bone marrow toxicity [159,160,161]

Other recognized risk factors for the development of pulmonary complications in SLE are overlapping autoimmune conditions. For example, the presence of antiphospholipid syndrome may lead to thrombocytopenia, pulmonary embolism, pulmonary hypertension, or pulmonary microthrombosis [162]. In addition, Lee et al. [66] performed an extensive literature review and discussed the increased frequency and severity of ILD-SLE in patients with overlap syndrome, such as systemic sclerosis, mixed connective tissue disease, and Sjögren’s syndrome. In these patients, ILD shows a high prevalence and is associated with worse survival outcomes [66]. Another retrospective multicenter study by Deneuville et al. [163] involving 89 SLE patients with ILD found that 47.2% were positive for anti-U1-RNP antibodies. Notably, 65.2% of these patients also had another connective tissue disease, such as Sjögren’s syndrome, systemic sclerosis, inflammatory myopathy, or rheumatoid arthritis. This overlap was associated with more severe pulmonary involvement, as indicated by impaired pulmonary function tests and the requirement for aggressive treatments like corticosteroids and immunosuppressants [163]. Besides ILD, other research groups have demonstrated that overlap syndromes increase the risk of PAH in SLE patients, particularly those with systemic sclerosis or mixed connective tissue disease features and anti-U1-RNP positivity [164,165,166]. These patients require proactive screening and may benefit from early referrals to specialized centers for pulmonary hypertension [164,165,166].

## 4. Research Method

This extensive literature review included all articles from April 2025 and earlier which were written in English, available in full-text, and published in peer-reviewed journals dealing with pulmonary manifestations of SLE. The article types included were prospective and retrospective cohort studies, randomized controlled clinical trials, relevant systematic reviews, meta-analyses, case reports, case series, and letters to the editor. We searched three electronic bibliographic databases (PubMed, Scopus, and Web of Science). The search was performed with the following combination of keywords: “systemic lupus erythematosus”, “pulmonary involvement, “pulmonary manifestations”, “pleuritis”, “lung parenchymal involvement”, “acute lupus pneumonitis”, “diffuse alveolar hemorrhage”, “interstitial lung disease”,” bronchiolitis obliterans organizing pneumonia”, “pulmonary hypertension”, “shrinking lung syndrome”, “infections”, and “chronic obstructive pulmonary disease”. We excluded studies that focused on primary pulmonary diseases or pulmonary diseases in the context of other autoimmune conditions. No restrictions were applied based on the publication date. Two independent authors (IM and GN) selected candidate studies based on the inclusion and exclusion criteria mentioned above. Finally, the two main authors (IM and GN) agreed on the final selection of the studies.

## 5. Conclusions

Pulmonary complications are common in patients with SLE and can significantly influence morbidity and mortality in affected subjects. For this reason, increasing physician awareness of lung involvement in SLE and improving the management of these conditions is pivotal. Pulmonary manifestations may occur in various forms, including pleuritis, acute and chronic lung parenchymal involvement, pulmonary arterial hypertension, and shrinking lung syndrome, each requiring distinct diagnostic and therapeutic approaches. Additionally, it is crucial to recognize that these manifestations often have a multifactorial origin. Indeed, such involvement frequently coexists with or is mimicked by infectious processes, cardiovascular or hemodynamic disturbances, and complex interactions among immune, thrombotic, and inflammatory pathways. Accordingly, rigorous differential diagnosis and a high index of suspicion for overlapping etiologies are crucial when interpreting pulmonary and pleural abnormalities in SLE cohorts. In this view, early recognition and appropriate treatment can prevent irreversible lung damage, improve quality of life, and reduce the risk of life-threatening complications. Moreover, lung manifestations may reflect disease activity or flare-ups, making them essential indicators for overall disease monitoring and management.

## Data Availability

No new data was generated or analyzed for this review article.
